# Graphene Aerogels With Spherical Pore Structure for Broad Frequency Regulation and Enhanced Low‐Frequency Response

**DOI:** 10.1002/advs.76348

**Published:** 2026-07-01

**Authors:** Liang Li, Jiale Yan, Gengping Wan, Changlong Du, Yubing Lv, Yongzhu Yan, Zhaoyang Li, Guizhen Wang

**Affiliations:** ^1^ Henan Key Laboratory of Biomarker Detection and Diagnosis for Neurodegenerative Diseases Shangqiu Normal University Shangqiu China; ^2^ State Key Laboratory of Tropic Ocean Engineering Materials and Materials Evaluation Center for Advanced Studies in Precision Instruments Hainan University Haikou China; ^3^ Center For New Pharmaceutical Development and Testing of Haikou Center for Advanced Studies in Precision Instruments Hainan University Haikou China

**Keywords:** dynamic frequency regulation, low‐frequency absorption, impedance matching, spherical‐pore structure

## Abstract

Conventional microwave absorbers, with their fixed operating frequencies and narrow bandwidths, are fundamentally limited in the face of advancing multifrequency radar systems. Strain tuning is highly favored as a dynamic regulation strategy because of its simple operation and rapid response. However, achieving broadband tunability and strong low‐frequency absorption in strain‐tunable microwave absorbers remains challenging, as even slight compression can lead to significant increases in conductivity. Herein, we develop a spherical‐pore‐structured graphene aerogel (SPGA) through a microbubble‐templating method. The spherical‐pore topology effectively suppresses strain‐induced percolation of conductive networks, rendering the electrical conductivity only weakly dependent on strain. SPGA achieves dynamic frequency tuning across 3.6–18 GHz while maintaining strong absorption under compressive strains of up to 70%. Simultaneously, it delivers enhanced low‐frequency absorption with an effective absorption bandwidth of 2.56 GHz, spanning 91% of the low‐frequency microwave spectrum. These results demonstrate the potential of topology‐guided structural design for strain‐tunable microwave absorption and suggest a viable route for the rational design of intelligent microwave absorbers.

## Graphical abstract

A graphene aerogel with a spherical‐pore structure is constructed via microbubble templating. This unique architecture suppresses strain‐induced percolation of conductive networks, enabling dynamic frequency tuning. It also delivers enhanced low‐frequency absorption with an effective absorption bandwidth of 2.56 GHz, covering 91% of the low‐frequency microwave spectrum. This work establishes a structure‐guided design strategy for intelligent microwave absorbers.

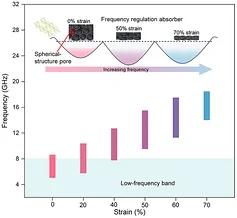

## 1 Introduction

Microwave absorbing materials (MAMs) are essential for mitigating electromagnetic (EM) pollution and enhancing stealth capabilities in applications ranging from portable electronics to next‐generation radar and wireless communication systems [1, 2, 3, 4, 5, 6, 7]. To address diverse and evolving application demands, a wide range of MAMs have been developed, including those based on magnetic loss (e.g., ferrites [8, 9], alloys [10], and magnetic nanoparticles [11]), dielectric loss (e.g., carbon materials [12, 13], conductive polymers [14], and ceramics [15, 16]), as well as their composites [17, 18, 19] and structural absorbers (e.g., metamaterials [20, 21] and chiral media [22]). These materials offer distinct advantages and can meet key performance requirements for microwave absorption (MA), including a broad effective absorption bandwidth and strong absorption capability [23, 24, 25, 26]. However, the rapid proliferation of frequency‐agile, multiband radar systems, together with the dense deployment of 5G/6G base stations and Internet‐of‐Things devices, has imposed far more stringent requirements. In such dynamically evolving EM environments, conventional fixed‐frequency absorbers are fundamentally limited, and there is an increasing demand for microwave absorbers that offer dynamically reconfigurable responses over a broad frequency range, with particular emphasis on robust low‐frequency performance.

Currently, achieving dynamic frequency tuning remains a significant challenge as it requires precise and reversible modulation of the complex permittivity or permeability of MAMs to overcome their intrinsic limitations. Besides, the structural integrity and operational stability must also be maintained under dynamic conditions to retain efficient MA performance. Much effort has been devoted to overcoming these limitations by developing stimuli‐responsive MAMs using electric fields [27], magnetic fields [28], and thermal stimuli [29]. Although these approaches demonstrate frequency‐shift capabilities, they suffer from several critical limitations: complex external control requirements [30], narrow tunable ranges (<3 GHz) [31], and compromised durability under cyclic operation [27]. Recently, we developed an innovative strain‐tuning strategy based on highly compressible carbon nanocoils/carbon foam, which enabled dynamic frequency regulation over a broad tuning range [22]. This strain‐based frequency‐tuning method offers a simple route toward dynamically frequency‐tunable MAMs. Nevertheless, in such systems, the underlying random porous skeleton tends to densify rapidly during compression, leading to sharp increases in electrical conductivity, deteriorated impedance matching, unsatisfactory low‐frequency absorption, and reduced operational stability under large or repeated strains.

Spherical‐pore architectures are known to effectively regulate electrical conductivity and possess excellent three‑dimensional isotropy, thereby ensuring stable structural and macroscopic performance [32, 33]. Inspired by this, we prepared a graphene aerogel with a spherical‐pore structure (SPGA) using a microbubble‐templating method, and the resulting SPGA exhibits tunable MA properties (Figure 1a). The spherical‐pore topology endows the SPGA with weak strain dependence of electrical conductivity and well‐preserved pore geometry under compression, enabling dynamic frequency tuning across 3.6–18 GHz, covering ≈91% of the measured MA frequency range, while maintaining strong absorption even under compressive strains of up to 70%. In addition, SPGA exhibits markedly enhanced low‐frequency absorption: the low‐frequency effective absorption bandwidth (EAB) reaches 2.56 GHz, up to 3.66 times larger than that of representative fixed‐frequency absorbers of comparable thickness reported in the literature (Figure 1b). The spherical‐pore architecture suppresses strain‐driven conductive percolation during compression, maintaining impedance matching and enabling broadband tuning across a wide strain range. Benefiting from this topology‑governed evolution, SPGA also exhibits robust Joule heating stability, characterized by only a slight rise in Joule heating temperature under 50% compressive strain. This is consistent with the observation that the Joule heating temperature does not increase sharply during compression, further confirming that the spherical‑pore structure suppresses excessive conductivity. Moreover, SPGA demonstrates excellent photothermal conversion performance. Mechanistically, these findings establish structural design as the enabler of broadband tunability in strain‑tunable absorbers, providing a practical path to adaptive EM protection and stealth materials.

**FIGURE 1:**
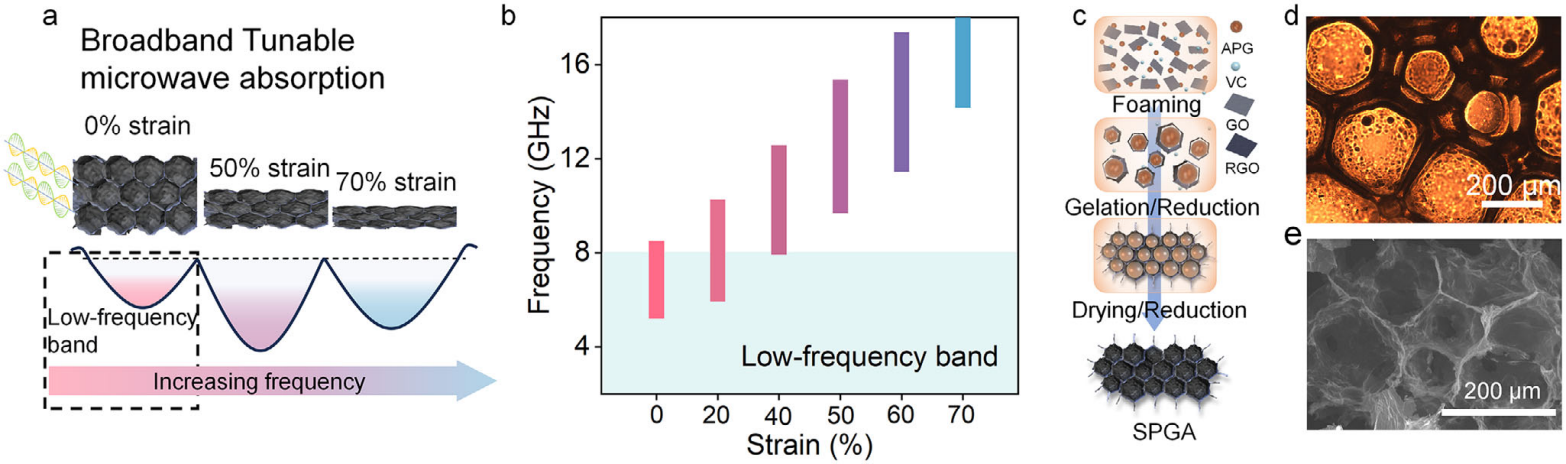
Design and fabrication of SPGA. a) Tunable microwave absorption. b) Frequency tuning and low‐frequency absorption under different compressive strains. c) Schematic illustration of the synthetic steps for fabricating SPGA. d) POM images of FGO with 80 µL of APG. e) SEM image of SPGA‐2.

## 2 Results and Discussion

### 2.1 Synthesis and Characterization of SPGA

As cost‐effective and environmentally friendly soft templates, APG‐generated microbubbles created under vigorous stirring serve as structural templates for constructing SPGAs (Figure 1c). Amphiphilic GO sheets spontaneously migrate to and assemble at the bubble interfaces, forming closed shells around the bubbles [34]. Subsequent in situ reduction/gelation fixes this bubble‐directed framework, and mild oven drying followed by thermal annealing yields lightweight SPGAs. By varying the APG content, the density and size of the bubbles, and hence the pore size and porosity can be tuned while preserving the overall pore arrangement (Figures S1–S3). The resulting aerogels are designated as SPGA‐x, with x = 1, 2, or 3 corresponding to APG additions of 40, 80, and 120 µL, respectively. Polarized optical microscopy confirms that GO sheets wrap around surfactant‐stabilized microbubbles rather than remaining homogeneously dispersed in the liquid phase (Figure 1d and Figure S4a–e). SEM imaging further reveals that the resulting SPGAs consist of a three‐dimensional network of interconnected graphene walls enclosing nearly spherical pores with a relatively narrow pore‐size distribution in the 100–200 µm range, separated by thin graphene walls (Figure 1e and Figure S4f). This contrasts sharply with conventional chemically reduced non‐microbubble‐templated graphene foam (GF), which shows densely stacked, randomly oriented nanosheets and largely featureless porosity (Figure S5). The bulk density of SPGA‐2 is ≈5.76 mg cm^−3^ (Figures S2 and S6), and the SPGA‐2 sample can rest on a flower, demonstrating its lightweight and highly porous nature.

The structural evolution from GO to SPGA was systematically verified by XRD, XPS, and Raman spectroscopy. GO exhibited a sharp diffraction peak at 11.02° (d = 0.802 nm), while unannealed RGO (URGO) showed a broadened peak at 23.09°, reflecting partial recovery of sp^2^ domains and *π–π* interactions. After thermal treatment, the SPGA peak shifted to 24.56°, corresponding to a reduced spacing of 0.361 nm. A transient peak at 11.53° in URGO, attributed to stacking of APG molecules at the gas–liquid interface, disappeared upon APG decomposition at 200°C, confirming its templating role (Figure 2a) [35]. XPS spectra revealed efficient removal of oxygen‐containing groups, with SPGA dominated by C–C bonding [36], while Raman spectra showed an increased I_D_/I_G_ ratio (0.99 to 1.32), evidencing enhanced conjugation and defect generation during reduction (Figure 2b,c).

**FIGURE 2:**
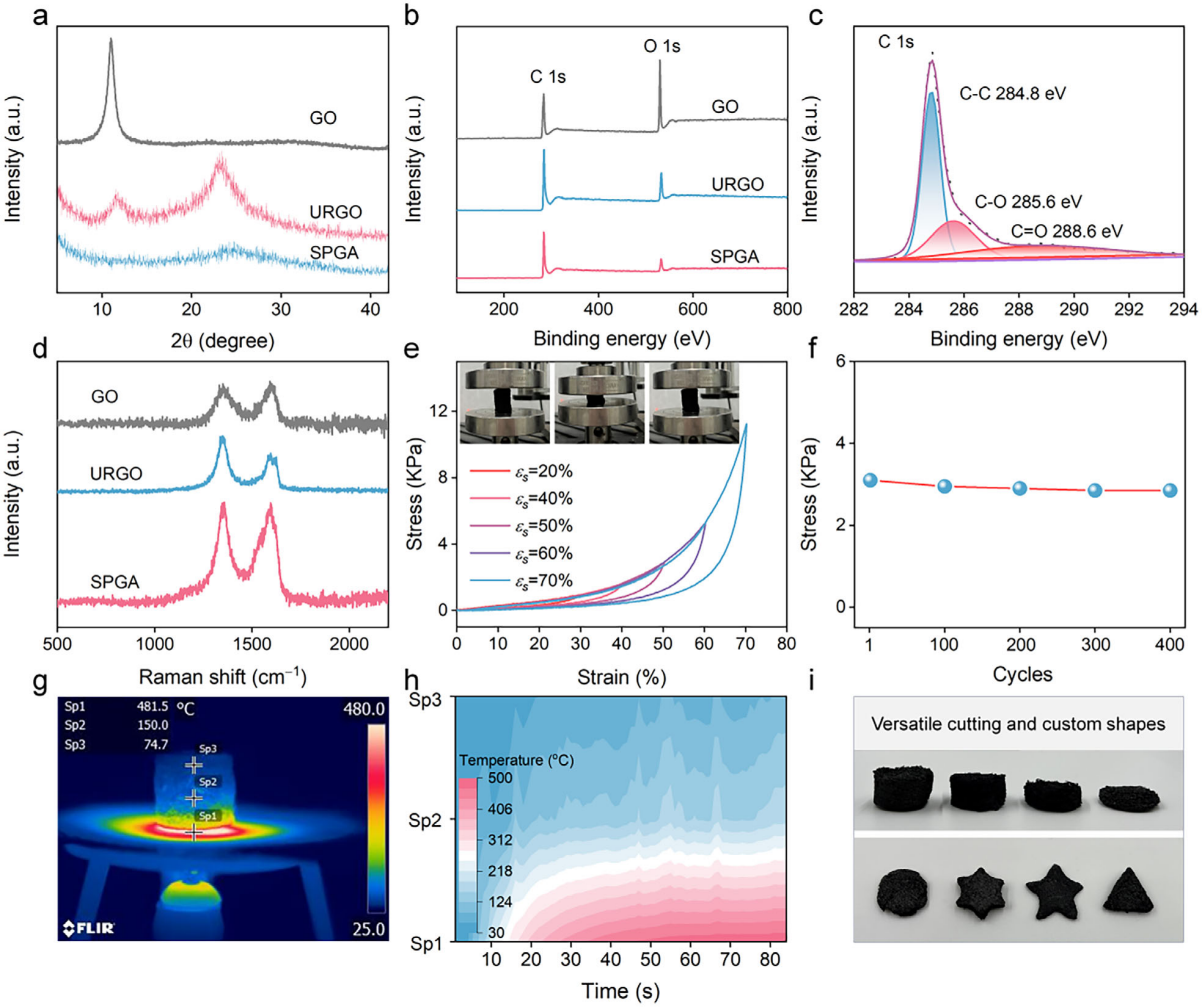
Structural characterization of SPGA. a) XRD patterns. b) XPS spectra of GO, URGO, and SPGA. c) C 1s XPS spectrum of SPGA. d) Raman spectra of GO, URGO, and SPGA. e) Stress–strain curves of SPGA‐2 under various compressive strains. Insets: Photographic images corresponding to compression–release testing. f) Stress retention during 400 cycles at 50% compressive strain. g) Infrared thermal image of SPGA heated by an alcohol lamp. h) Corresponding temperature–time curves of the test points. i) Photographic images of SPGA cut into custom shapes.

The spherical‐pore nanowall network of SPGA provides exceptional mechanical and functional performance (Figure 2d–f). The aerogel recovers rapidly after unloading from compressive strains of 20–70% and retains >92% of its maximum stress after 400 compression cycles at 50% strain. Even under extreme conditions (70% strain, 50 cycles), it maintains 95% height recovery and 92% stress retention (Figure S7). A 30 mm‐thick SPGA further exhibits superior thermal insulation, maintaining top and bottom surface temperatures of 74.7 and 481.5°C, respectively, under flame exposure (Figure 2g,h). Importantly, SPGA exhibits excellent machinability. It can be cut and shaped into complex geometries while preserving structural integrity, allowing precise thickness control (Figure 2i). Since reflection loss (RL) strongly depends on coating thickness, this dimensional tunability enables fine optimization of MA performance, underscoring its promise for scalable and adaptable EM protection applications.

### 2.2 Static EM Performance: From EMI Shielding to MA

The microbubble‐templated restructuring of GO not only changes the geometry and porosity of the aerogel but also fundamentally reorganizes the conductive network and EM wave (EMW) propagation pathways. To clarify the static EM behavior, the reduced GF and SPGAs were compared. At a thickness of 2 mm, GF exhibits an EMI shielding effectiveness (EMI SE) of ≈15.8 dB (Figure 3a,b and Figure S8), dominated by strong reflection at the air–material interface. Its densely stacked, highly conductive layered architecture induces severe impedance (Z) mismatch, causing most incident EMWs to be reflected rather than absorbed. In contrast, the microbubble‐templated SPGAs undergo a clear transition from reflection‐dominated shielding to efficient MA. Among them, SPGA‐2 shows optimized performance: at a thickness of 3.3 mm, it reaches an RL_min_ of −59.44 dB with a maximum EAB (EAB_max_) of 7.44 GHz (Figure 3c). SPGA‐1 and SPGA‐3 also show a similar reflection‐to‐absorption transition, but with slightly lower performance. At the same thickness, their RL_min_ values are −14.78 and −52.84 dB, with corresponding EAB_max_ values of 4.48 and 6.08 GHz (Figure 3d–f). Furthermore, within the thickness range of 2.1–4.5 mm, the RL_min_ values of SPGAs are all below −10 dB (Figure 3g and Figure S9). These results indicate that incident EMWs can penetrate the air–material interface and undergo efficient absorption within the porous network, rather than being predominantly reflected [12, 37]. Remarkably, SPGA‐2 outperforms many state‐of‐the‐art absorbers (Figure 3h and Table S1) [38, 39, 40, 41, 42, 43, 44, 45], which highlights the critical role of microbubble‐driven microstructure engineering in tailoring EM functionality.

**FIGURE 3:**
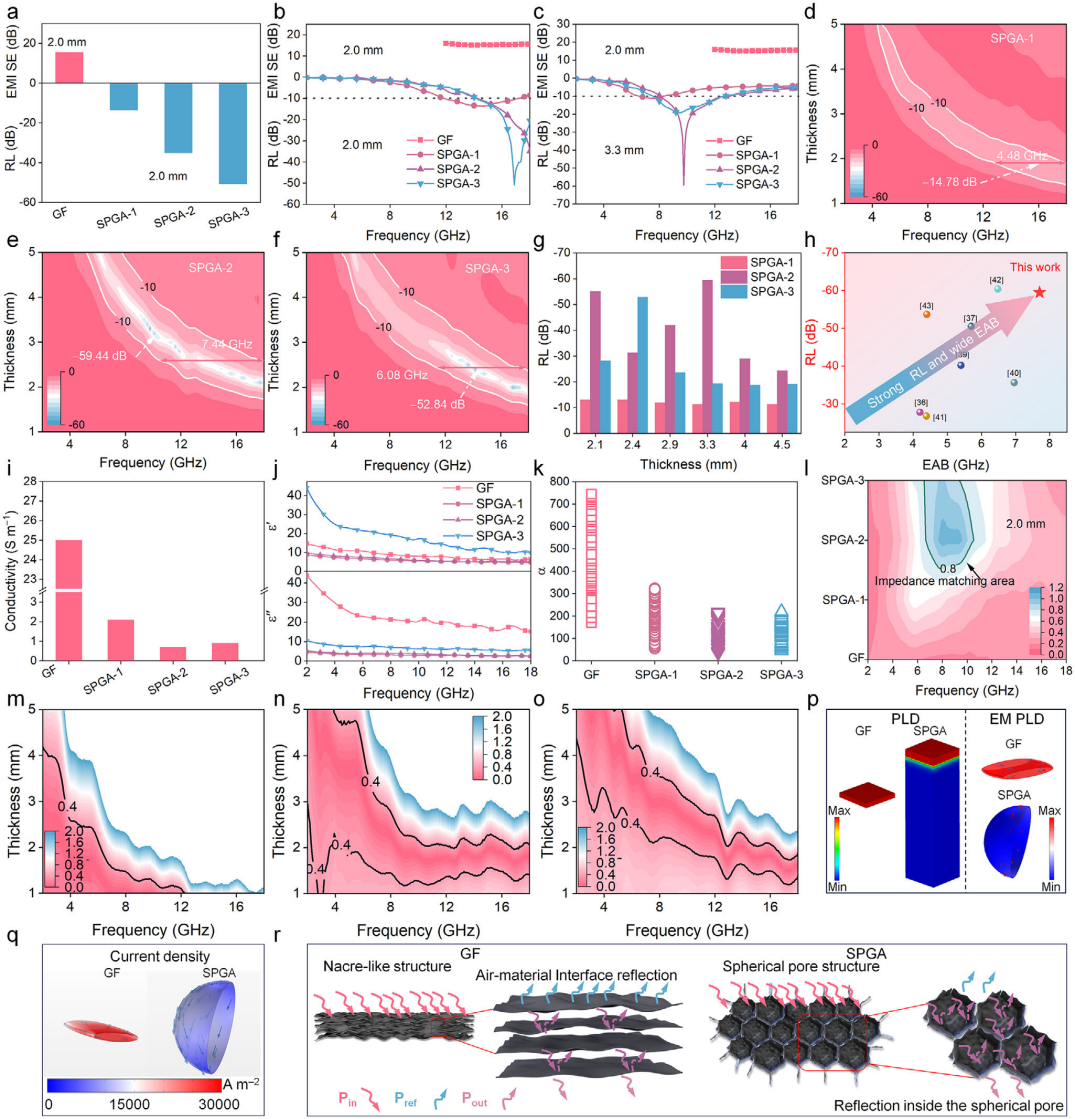
EM performance and mechanism of GF and SPGA. a,b) EM performance of GF and SPGA at a thickness of 2 mm. c) EMI shielding and RL_min_ of GF and SPGA. d–f) 2D RL mappings of SPGA‐1, SPGA‐2, and SPGA‐3, respectively. g) RL_min_ at different thicknesses. h) Comparison of RL_min_ and EAB_max_ among previously reported absorbers. i–k) Conductivity, *ε′* and *ε″*, and *α* of GF and SPGA. l) 2D Z mapping of GF and SPGA. m–o) Impedance matching characteristics of SPGA‐1, SPGA‐2, and SPGA‐3, respectively. p) Simulated PLD distribution at 9.76 GHz. q) Current‐density distribution. r) Schematic representation of EM mechanisms of GF and SPGA.

The structural effects on EMW responses were further probed by CST‐based radar cross‐section (RCS) simulations (Figures S10 and S11) [46]. While GF and SPGA‐1 show limited suppression of backscattered signals, SPGA‐2 and SPGA‐3 display superior attenuation, consistent with their improved *Z* matching and absorption efficiency. This highlights that the spherical‐pore structure in SPGA not only enhances bulk absorption but also effectively suppresses backscattering, a key requirement for stealth and EM protection applications.

The underlying reason for this transformation can be understood from the conductivity, dielectric parameters, and Z behavior. All samples show *µ′* ≈ 1 and *µ″* ≈ 0 over the measured band (Figure S12), indicating that dielectric loss dominates. Relative to GF, the spherical‐pore‐structured SPGAs exhibit moderate conductivity due to reduced overlap and reorientation of graphene walls (Figure 3i), which in turn brings *ε″* and the attenuation constant (*α)* into an optimal range (Figure 3j). This reduction in *σ* and *ε″* alleviates the severe *Z* mismatch of GF while still maintaining sufficient loss for effective attenuation (Figure 3k). Cole–Cole plots (Figure S13) confirm that conduction loss is prevalent at low frequencies, whereas polarization/relaxation processes dominate at higher frequencies, providing multiple channels for EM energy dissipation [47]. Foaming changes the Z of the material from mismatched to matched. As a result, the *Z* of SPGAs approaches unity over a broad band (Figure 3l–o and Figure S14), indicating the transition from a reflection‐dominated regime of GF to a Z‐matched absorption regime of SPGAs.

Simulations further highlight the role of topology in EMW propagation. CST and COMSOL calculations show that in GF, the power loss density (PLD) is concentrated near the front surface, leading to strong reflection and shallow penetration (Figure 3p). Moreover, the layered structure exhibits a higher and more localized PLD near the front surface than that in the spherical‐pore structure. In SPGA‐2, however, the spherical‐pore network extends the EMW path length and redistributes PLD more uniformly throughout the bulk, enabling deeper penetration and more complete attenuation. Current‐density and field‐distribution maps (Figure 3q) reveal that the layered architecture of GF supports intense surface currents and severe *Z* mismatch, whereas the spherical‐pore topology of SPGA promotes diffuse currents, improved *Z* matching, and strong internal dissipation.

In summary, GF consists of densely stacked RGO nanosheets forming a highly conductive layered architecture, which induces severe *Z* mismatch and reflection‐dominated shielding (Figure 3r). After foaming, SPGA transforms into a 3D interconnected porous network, with microbubble‐induced nanosheet orientation lowering conductivity and optimizing *Z* matching. This structural reorganization enables incident EMWs to penetrate the aerogel and undergo multiple scattering/reflection and dielectric/conductive losses, leading to efficient attenuation. Importantly, the foaming process not only reconfigures the microstructure for tunable EM performance but also simplifies fabrication by eliminating freeze‐drying, thereby enabling scalable production. These advantages make SPGA a highly promising platform for next‐generation microwave absorbers and EM protection systems.

### 2.3 Tunable Absorption Frequency Under Compressive Strain

For adaptive microwave absorbers, it is important not only to achieve excellent performance under a fixed condition but also to controllably adjust the absorption frequency under different working conditions. Therefore, we systematically investigated the dependence of the MA properties of the SPGAs on sample thickness and compressive strain. On this basis, we constructed a thickness–strain map that links these structural parameters to the tunable frequency range and effective absorption bandwidth.

SPGA‐1 exhibits negligible absorption at high strains due to excessive conductivity and poor *Z* matching, restricting its effective absorption to low frequencies with narrow bandwidths (Figures S16–S19). In contrast, SPGA‐2 demonstrates outstanding compression‐tunable performance across thicknesses of 4–8 mm (Figure 4 and Figure S20). At an initial thickness of 4.7 mm, SPGA‐2 reaches an RL_min_ of −66.24 dB at 50% strain (Figure 4a). Furthermore, the tunable absorption frequency ranges are 6.48–18 GHz, 5.52–18 GHz, and 4.32–13.52 GHz for thicknesses of 4, 4.7, and 6 mm, respectively, collectively spanning the C, X, and Ku bands (Figure 4b–d,g,h and Figure S21). Remarkably, with a 7 mm initial thickness, SPGA‐2 achieves an ultra‐wide tunable absorption band spanning 3.6–18 GHz, encompassing the S, C, X, and Ku bands and covering 91% of the tested spectrum (Figure 4e,j). With an 8 mm initial thickness, SPGA‐2 achieves an ultra‐wide tunable absorption band encompassing the S, C, and X bands (Figure 4f). At 4 mm, SPGA‐2 also maintains excellent bandwidth, with an EAB of 6.32 GHz at 40% strain (Figure 4h). SPGA‐3 exhibits similar strain‐responsive behavior across thicknesses of 3.6–8 mm (Figures S21 and S22). At 3.6 mm, it achieves an RL_min_ of −64.23 dB under 20% strain and a tunable absorption bandwidth of 11.36 GHz (6.64–18 GHz) across the 0–70% strain range, along with an EAB_max_ of 5.92 GHz at 40% strain (Figure S22a,g). For thicker samples (6–8 mm), the absorption frequency can be shifted across the S, C, and X bands, demonstrating flexible band‐selective tunability (Figure 4i–k).

**FIGURE 4:**
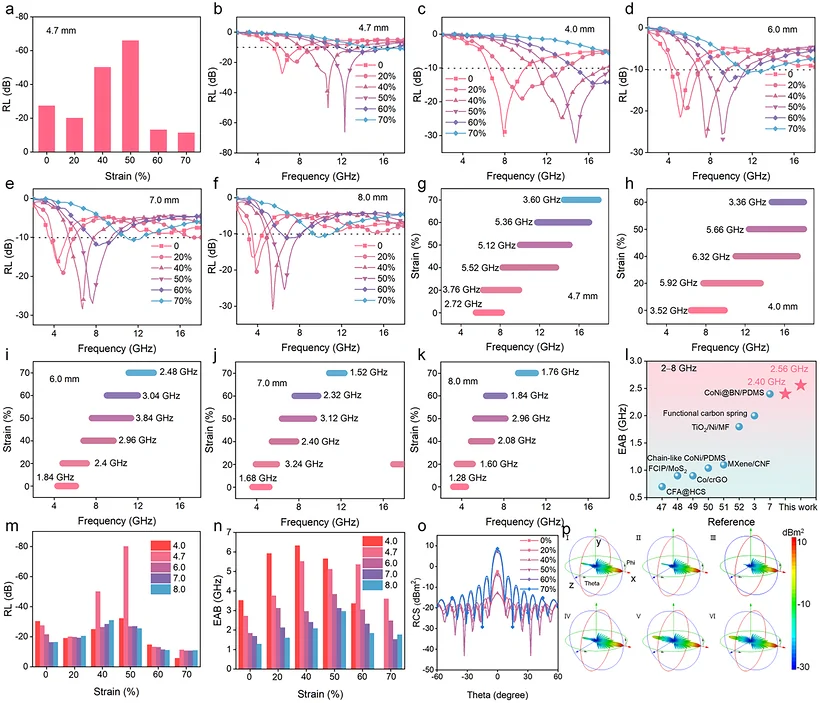
Dynamic frequency tuning properties of SPGA‐2. a,b) RL maps and RL curves of SPGA‐2 with an initial thickness of 4.7 mm under different compressive strains. c–f) Strain‐dependent RL curves at initial thicknesses of 4, 6, 7, and 8 mm, respectively. g–k) EAB traces at initial thicknesses of 4.7, 4, 6, 7, and 8 mm, respectively. l) Comparison of low‐frequency EAB among previously reported absorbers. m,n) Summary of strain‐dependent RL and corresponding EAB values at 4–8 mm. o) RCS curves for the PEC layer covered with SPGA‐2 under different compressive strains. p) CST simulation results of I–VI) 3D RCS plots for the PEC layer covered with SPGA‐2 at 0%, 20%, 40%, 50%, 60%, and 70% compressive strains.

An 8 mm sample compressed by 50% achieves an EAB of 2.56 GHz (Figure 4k), outperforming most reported low‐frequency compression‐tunable absorbers and delivering an EAB 3.66 times larger than that of previously reported fixed‐frequency absorbers in the same frequency region (Figure 4l). Beyond low‐frequency advantages, SPGA also excels in overall absorption efficiency and thin‐layer performance. A comprehensive comparison (Figure 4m,n and Figure S23) confirms that SPGA‐2 and SPGA‐3 outperform conventional absorbers at minimal thicknesses. SPGA‐2 achieves RL values below −30.36 dB and EAB >3.52 GHz at only 4 mm, while SPGA‐3 maintains RL values below −19.70 dB and EAB >3.84 GHz at 3.36 mm. Importantly, SPGA‐2 delivers RL values below −60 dB at moderate compression (20–50%), outperforming most reported microwave absorbers (Figure 4h) [3, 7, 48, 49, 50, 51, 52, 53, 54, 55]. These results highlight the unique advantages of spherical‐pore architectures for combining wide frequency tunability with high absorption efficiency.

The practical impact of this tunability is corroborated by RCS simulations. When a PEC substrate is covered by SPGA‐2, the monostatic RCS is significantly reduced over a wide angular range (0° ± 60°) for strains up to ≈60% (Figure 4o,p and Figure S24), in excellent agreement with the *Z* and RL design map. At higher compressive strains, the RCS reduction weakens, consistent with the onset of excessive conductivity and degraded matching.

The frequency‐tunable MA performance of SPGA‐2, combining low RL values, broad EAB, and small thickness, can be attributed to four key factors:

(i) Conductivity. SPGA‐2 exhibits remarkably weak strain dependence of electrical conductivity (Figure 5a and Equation S11). Its spherical‐pore framework architecture suppresses the excessive growth of conductive networks under compression. Specifically, edge‐contact–dominated interfaces yield only gradual contact expansion under strain, and preferential inter‐sheet sliding prevents the formation of bending‐mediated conductive bridges. As a result, parallel low‐resistance percolation pathways are not readily formed during pore collapse. Even at 70% strain, conductivity increases by only ≈170%, significantly lower than that in freeze‐dried RGO aerogels (Figure S15b). This weak conductivity dependence prevents abrupt deterioration of *Z* matching, enabling stable and broad frequency tunability.

**FIGURE 5:**
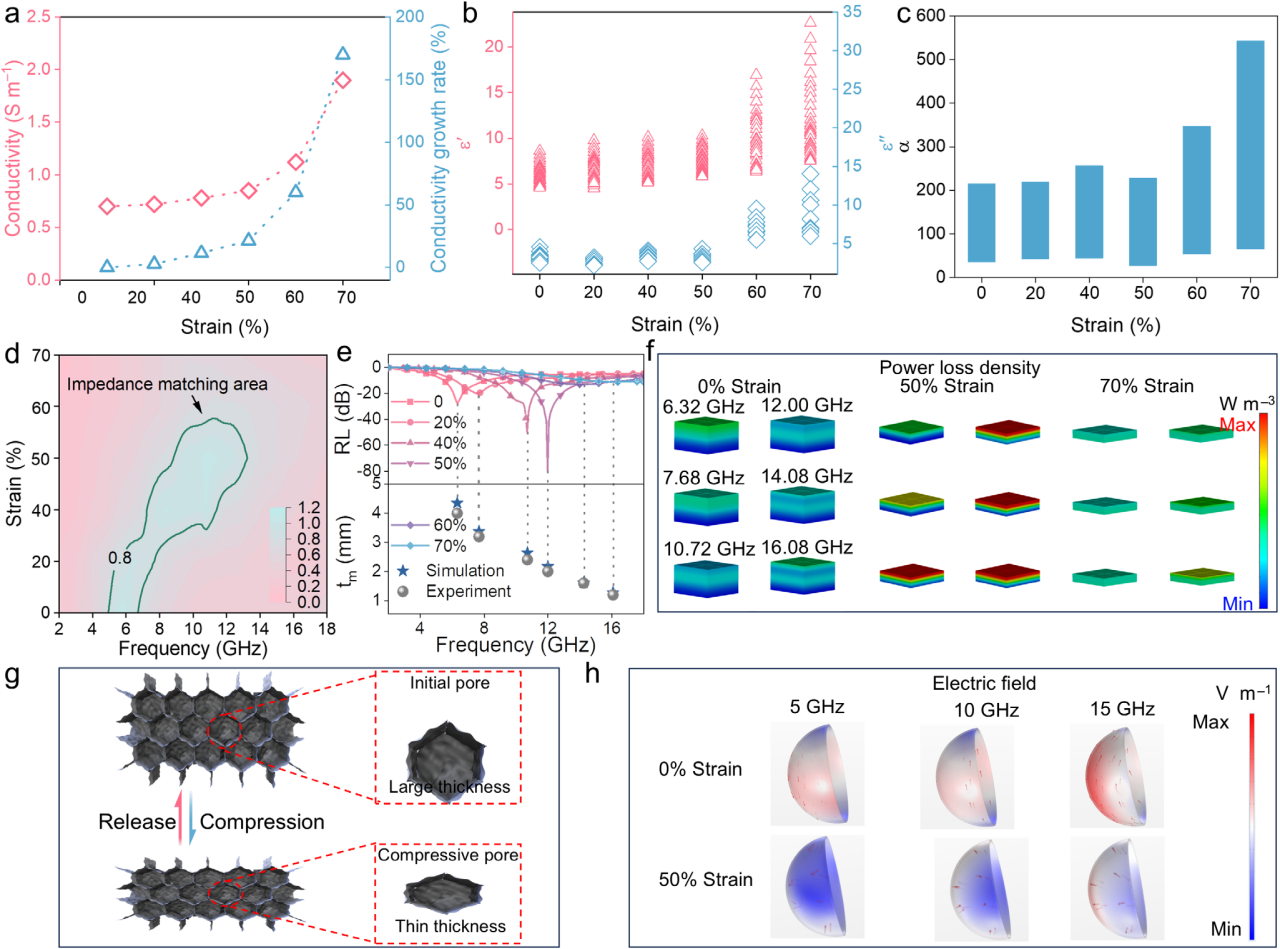
Mechanism for dynamic frequency modulation of SPGA‐2. a) Conductivity. b) *ε′* and *ε″*. c) *α*. d) *Z* values. e) RL and *t_m_* values based on the *λ*/4 model under different compressive strains. f) Simulated PLD distributions at 6.32, 7.68, 10.72, 12, 14.08, and 16.08 GHz under compressive strains of 0%, 50%, and 70%. g) Pore structure before and after compression. h) Electric‐field polarization direction and electric‐field intensity simulations of SPGA‐2 at 0% and 50% compressive strain.

(ii) EM parameters. According to transmission line theory, absorption frequencies can be dynamically tuned via dielectric parameters. As shown in Figure 5b, *ε′* and *ε″* of SPGA‐2 increase only slightly below 60% strain, consistent with the limited conductivity changes (Figure 5a). At higher strains (60–70%), enhanced conductive pathways promote stronger conduction loss and interfacial polarization, leading to larger *ε′* and *ε″*. As a dielectric absorber, SPGA‐2 maintains constant magnetic permeability (*µ′* ≈ 1, *µ″* ≈ 0) (Figure S25). The *α* follows conductivity and permittivity trends, remaining stable below 60% strain and rising at higher compression (Figure 5c). Importantly, SPGA‐2 preserves excellent *Z* matching under moderate compression (Figure 5d and Figure S27), ensuring efficient EM penetration and energy dissipation [56].

(iii) Matching thickness. Frequency‐selective absorption is also modulated by thickness according to the *λ*/4 principle (Equation S12) [22]. Experimentally, the absorption peak frequency (*f_m_*) shows a clear blueshift with increasing strain (Figure 5e and Figure S28), closely following Equation S12. Slight deviations arise from the coexistence of multiple loss pathways, including conduction, polarization, multiple reflections, and scattering. CST simulations of PLD confirm this trend (Figure 5f, Figure S29, and Table S2). As compression increases, PLD_max_ shifts progressively from lower to higher frequencies (6.32–16.08 GHz), in agreement with experimental results. PLD is distributed throughout the upper and inner absorber regions, indicating deep EM penetration and extended propagation paths that enhance energy dissipation. These findings establish compression‐controlled thickness adjustment as a reliable mechanism for dynamic frequency tuning.

(iv) Adjustable pore structure. The spherical‐pore network provides transmission pathways that facilitate multiple scattering and reflection, while its geometry can be precisely modulated by strain (Figure 5g). COMSOL simulations (Figure 5h) reveal that compression‐induced pore evolution alters both the electric‐field distribution and polarization direction. At 5, 10, and 15 GHz, spanning conduction‐dominated and polarization‐dominated regimes, compression systematically reduces pore size, intensifies local field redistribution, and shifts responses toward higher frequencies. This confirms that pore geometry directly governs energy dissipation efficiency and tunability.

The unique combination of weak strain dependence of conductivity, stable EM parameters under moderate compression, *λ*/4‐guided thickness modulation, and dynamically adjustable pore geometry endows SPGA‐2 with exceptional strain‐responsive MA performance. Supported by both experimental results and simulations, this synergistic mechanism enables precise frequency tuning across broad bands, underscoring the promise of spherical‐pore aerogels for adaptive stealth and real‐time EMI mitigation.

In addition to its microwave absorption capability, SPGA also exhibits stable Joule heating and efficient photothermal conversion, as detailed in Figure S30–S32. Briefly, the spherical‐pore topology endows the aerogel with strain‑insensitive heating output, preventing thermal runaway under mechanical deformation. At 2.0 V, the saturation temperature increases by only ≈24°C at 50% strain and by ≈85°C even at 70% strain, demonstrating that large mechanical deformation does not trigger catastrophic overheating. Meanwhile, under simulated solar illumination, SPGA delivers a fast photothermal response (∼12°C s^−1^) and stable, intensity‑controlled heating, enabling outdoor de‑icing within minutes. These additional functionalities highlight the potential of the spherical‑pore architecture for multi‑field adaptive thermal management. SPGA is promising for smart radomes in cold, humid environments, where stable Joule heating and excellent photothermal conversion performance enable rapid de‑icing and defogging, and tunable microwave absorption provides adaptive electromagnetic protection.

## 3 Conclusions

In summary, we demonstrate that the spherical‐pore‐structured graphene aerogel (SPGA) addresses key longstanding limitations of conventional microwave absorbers, simultaneously achieving broad dynamic frequency tunability and enhanced low‐frequency absorption. Leveraging its spherical‐pore structure, SPGA enables reversible absorption frequency tuning across 3.6–18 GHz, alongside an exceptionally broad low‐frequency effective absorption bandwidth of 2.56 GHz, which is 3.66 times larger than that of previously reported fixed‐frequency absorbers operating in the low‐frequency region. Remarkably, it maintains robust microwave absorption even under an extreme compressive strain of up to 70%, while the spherical‐pore architecture suppresses strain‐induced conductive network densification, ensuring stable electrothermal performance. Furthermore, SPGA supports ultrafast photothermal conversion with a rate of up to 12°C s^−1^. This work provides a structural design strategy for developing intelligent microwave absorbers with broad frequency tunability, enhanced low‐frequency response, and deformation‐tolerant functional stability.

## Author Contributions

**Changlong Du**: methodology, software. **Zhaoyang Li**: formal analysis, visualization, funding acquisition, project administration, writing – review and editing. **Liang Li**: conceptualization, methodology, writing – original draft, funding acquisition, project administration. **Gengping Wan**: project administration, investigation. **Yubing Lv**: validation, resources. **Yongzhu Yan**: investigation, methodology. **Guizhen Wang**: funding acquisition, writing – review and editing, supervision, project administration. **Jiale Yan**: data curation, formal analysis.

## Conflicts of Interest

The authors declare no conflicts of interest.

## Supporting information




**Supporting File**: advs76348‐sup‐0001‐SuppMat.docx.

## Data Availability

The data that support the findings of this study are available from the corresponding author upon reasonable request.
