# Supplementary material for: Graphene Aerogels With Spherical Pore Structure for Broad Frequency Regulation and Enhanced Low‐Frequency Response
Source: Adv Sci (Weinh). 2026 Jul 1;13(53):e76348. doi: 10.1002/advs.76348 (PMC13337031; doi:10.1002/advs.76348)
Supplement: Supplementary file 1 [file ADVS-13-e76348-s001.docx]

**Supporting Information**

**Graphene Aerogels with Spherical Pore Structure for Broad Frequency Regulation and Enhanced Low-Frequency Response**

*Liang Li, Jiale Yan, Gengping Wan*, Changlong Du,* *Yubing Lv, Yongzhu Yan, Zhaoyang Li*, and Guizhen Wang****

L. Li, J. Yan, Y. Lv, Z. Li

Henan Key Laboratory of Biomarker Detection and Diagnosis for Neurodegenerative Diseases, Shangqiu Normal University, Shangqiu China.

E-mail: [liliangabc@sqnu.edu.cn](mailto:liliangabc@sqnu.edu.cn), [13938512496@163.com](mailto:13938512496@163.com), lyb8449@163.com, [lizhaoyang@sqnu.edu.cn](mailto:lizhaoyang@sqnu.edu.cn)

G. Wan, C. Du, Y. Yan, G. Wang

State Key Laboratory of Tropic Ocean Engineering Materials and Materials Evaluation, Center for Advanced Studies in Precision Instruments, Hainan University, Haikou, China.

Center for New Pharmaceutical Development and Testing of Haikou, Center for Advanced Studies in Precision Instruments, Hainan University, Haikou, China.

E-mail: [wangengping001@163.com](mailto:wangengping001@163.com), [duchanglong@hainanu.edu.cn](mailto:duchanglong@hainanu.edu.cn), [yzyancam@hainanu.edu.cn](mailto:yzyancam@hainanu.edu.cn), wangguizhen0@hotmail.com

1. **Experimental Section**
   1.1. **Chemicals.**

Graphene oxide (GO) was provided by Carbene Technology Co., Ltd., China. Ascorbic acid (VC) was bought from Shanghai Guanghua Technology Co., Ltd, China. Alkyl Polyglucoside (APG) was purchased from Jiangsu Wanqi Biotechnology Co., Ltd, China. This thermochromic ink was purchased from Shenzhen Huancai Color Changing Technology, and its color-changing temperature is 65 °C. All chemicals were used without further purification.

1.2. **Preparation of SPG Hydrogel**

0.16 g GO was added to 20 ml DI water and stirred at 70 ℃ for 40 mins. 0.16 g ascorbic acid (VC), and different content of APG were then added to the GO solution and stirred for 3 min. The prepared foaming RGO dispersion was reacted at 80 ℃ for 12 h to obtain SPG hydrogel.

1.3. **Preparation of SPGA**

The prepared SPGA gel was frozen completely at −18 °C for about 4−6 h and then completely thawed at 60 °C in air. The SPGA was washed with DI water 1−3 times to remove most residual ascorbic acid and then dried at 60 °C in air for about 12 h to obtain SPGA. The SPGA was annealed at 200 °C in air for 4 h to obtain the SPGA. The obtained SPGA with APG content of 40 ul, 80 ul, and 120 ul were named SPGA-1, SPGA-2 and SPGA-3, respectively. In contrast, a RGO aerogel without APG was prepared using the same method.

2. **Characterization**
   2.1. **Apparatus**

The Polarized Optical Microscopy (POM) observations were performed with the BX 61 polarizing microscope (Olympus, Japan), using the Axiocam 506 color camera. The liquid samples were loaded between glass slides. The morphology of SPGA was examined by scanning electron microscope (SEM, Thermo Scientific Verios G4 UC). X-ray diffraction (XRD; Smart Lab II) was applied to analyze the crystal structures. X-ray photoelectron spectroscopy (XPS) was performed utilizing an AXIS SUPRA using an Al Kα anode. Raman spectra were recorded by Via Reflex (Renishaw) with a laser wavelength of 532 nm. The compression experiments were measured by an electronic universal testing machine (ETM series, WANCE) at a 10 mm min^−1^ strain rate. The temperature change of the center point of the composite film was monitored with a thermal imaging camera (E60, FLIR).

2.2. **EM Performance Test**

Electromagnetic parameters were obtained using an Agilent N5230A vector network analyzer in the frequency of 8.2–12.4 GHz (X-band) at room temperature.

The scattering parameters (*S_11_* and *S_21_*) in the X-band and K-band were recorded to calculate the coefficients of reflection (*R*), absorption (*A*) and transmission (*T*), total EMI SE (*SE_T_*), absorption (*SE_A_*), and reflection (*SE_R_*) using the following equations:

| $R=\left\vert S_{11} \right\vert^{2}$, $T=\left\vert S_{21} \right\vert^{2}$ | (S1) |
| --- | --- |
| $A=1-R-T$ | (S2) |
| ${SE}_{R}=-10\log\left( 1-R \right), {SE}_{A}=-10\log\left( \frac{T}{1-R} \right)$ | (S3) |
| ${SE}_{T}={SE}_{R}+{SE}_{A}+{SE}_{M}$ | (S4) |

Among them, when SE_T_ > 15 dB, SEM can be usually ignored.

The EM parameters were determined using a Ceyear 3672B-S vector network analyzer in the frequency range of 2–18 GHz according to the coaxial line method. A binder of paraffin is used to immobilize the aerogel because the aerogel can quickly recover its original shape after stress release and paraffin exhibits low electromagnetic parameters approximating those of air. The test sample was fabricated by vacuum-impregnating the aerogel with melted paraffin. After the aerogel is filled with paraffin, a certain pressure is applied to obtain the sample with the desired strain. The resulting foam was cut into standard coaxial rings with an outer diameter of 7.00 mm and an inner diameter of 3.04 mm (2.00 mm thick).

The reflection loss values of as-prepared samples were researched according to the transmission line theory:

$Z_{in}=Z_{O}\sqrt{\frac{\mu_{r}}{\varepsilon_{r}}}\tan h\left( j\frac{2\pi fd}{c}\sqrt{\mu_{r}\varepsilon_{r}} \right)$

(S5)

$$RL=20log\left| \frac{Z_{in}-Z_{0}}{Z_{in}{+Z}_{0}} \right|$$

(S6)

where *Z_in_* is the input impedance of the absorber, Z_0_ the impedance of free space, *μ_r_* the relative complex permeability ($\mu_{r}=\mu'-i\mu"$), *ε_r_* the complex permittivity ($\varepsilon_{r}=\varepsilon'-i\varepsilon"$), *f* the frequency of microwaves, *c* the velocity of light, and d the thickness of the absorber.

The $\varepsilon'$ and $\varepsilon"$according to Debye theory:

$$\varepsilon'=\varepsilon_{\infty}+\frac{\varepsilon_{s}-\varepsilon_{\infty}}{1+\left( \omega\tau_{0} \right)^{2}}$$

(S7)

$$\varepsilon"=\frac{\omega\tau_{0}\left( \varepsilon_{s}-\varepsilon_{\infty} \right)}{1+\left( \omega\tau_{0} \right)^{2}}+\frac{\sigma}{\omega\varepsilon_{0}}$$

(S8)

where, $\varepsilon_{\infty}$ is the relative dielectric permittivity in the high-frequency limit, *𝜏* is the polarization relaxation time, 𝜔 is the angular frequency, $\varepsilon_{s}$ is the static permittivity, $\varepsilon_{0}$ is the dielectric constant of vacuum, *σ* is the electrical conductivity.

The attenuation constant (*α*) was calculated by the following equation:

$$\alpha=\frac{\sqrt{2}\pi f}{c}\times\sqrt{\left( \mu"\varepsilon"-\mu'\varepsilon' \right)+\sqrt{\left( \mu"\varepsilon"-\mu'\varepsilon' \right)^{2}+\left( \mu"\varepsilon'+\mu'\varepsilon" \right)^{2}}}$$

(S9)

The Cole-Cole plots are obtained according to the equation S6:

$$\left( \varepsilon^{'}-\frac{\varepsilon_{s}+\varepsilon_{\infty}}{2} \right)^{2}+\left( \varepsilon^{''} \right)^{2}=\left( \frac{\varepsilon_{s}+\varepsilon_{\infty}}{2} \right)^{2}$$

(S10)

Electrical conductivity was calculated according to the following equations, where L is the length of the test sample, S is the cross-sectional area of sample and R is the resistance of sample).

$$\sigma= \frac{L}{SR}$$

(S11)

The matching thickness ($t_{m}$) according to quarter-wavelength (*λ*/4) theory:

$$t_{m}=\frac{n\lambda}{2}=\frac{nc}{4f_{m}\sqrt{\left| \varepsilon_{r}\mu_{r} \right|}} \left( n=1, 3,5\ldots. \right)$$

(S12)

Where *Z_in_* is the impedance of microwave absorber and *Z_0_* is the impedance of free space. When the *Z* value is close to 1, microwave can enter the absorber easily and then be converted to thermal or other energy.

2.3. **Simulation**

The CST Studio Suite 2020 was used for simulating the radar cross-section (RCS) of the microwave absorber at a frequency of 9.76 GHz. According to the widely accepted metal back model, the simulation model of the specimens was established as a square (200 mm × 200 mm) with dual layers. In detail, the bottom set as 2.0 mm is the perfect conductive layer (PEC), and the upper set as 3.3 mm signifies the absorbing layer. The PEC and the absorbing model plate were placed on the X–O–Y plane and linear polarized plane electromagnetic waves incident from the positive direction of the Z-axis to the negative direction of the Z-axis. Simultaneously, the direction of electric polarization propagation is along the X-axis. Open (add space) boundary conditions were employed in the x, y, and z directions. Generally, the scattering directions of RCS value can be determined by theta and phi in spherical coordinates, which can be described below:$\sigma=10log(\left( \frac{4\pi S}{\lambda^{2}} \right){\left| \frac{E_{S}}{E_{i}} \right|)}^{2}$

Here, *S, λ, E_s_* and *E_i_* are the area of the target object simulation model, the wavelength of electromagnetic wave, the electric field intensity of scattered wave, and the incident wave, respectively.

The Computer Simulation Technology (CST) Microwave Studio was employed to analyze the power loss density (PLD) distribution of the structure and SPGA aerogel under various strains. To reduce computational cost, a rectangular model with a cross-sectional area of 4 mm × 4 mm was selected for the simulations. In the analysis of the effects of different structures on microwave absorption, the sample thicknesses were set to 2 mm and 58 mm. Incident microwaves at a frequency of 9.76 GHz were excited from the top and injected into the material along the Z-axis. Under different strain levels of 0%, 20%, 40%, 50%, 60%, and 70%, the sample thicknesses were 4.78 mm, 3.82 mm, 2.87 mm, 1.9 mm, and 1.43 mm, respectively. For frequencies of 6.32 GHz, 7.68 GHz, 10.72 GHz, 12.00 GHz, 14.08 GHz, and 16.08 GHz, incident microwaves were excited from the top and injected into the SEGO along the Z-axis. The excitation ports were set to waveguide excitation. The boundary conditions for the modes were as follows: the X-axis as an electric boundary (Et = 0), the Y-axis as a magnetic boundary (Ht = 0), and the Z-axis as an open boundary. The electromagnetic parameters of SPGA used in the simulation were obtained from Ceyear 3672B-S vector network analyzer test.

The electric-field polarization directions and electric-field intensities were simulated using COMSOL Multiphysics 5.6 with the RF module. The simulation models of the sample were obtained with a periodic unit model. In the simulation model, the structural feature size of samples was magnified by 100 times. The incident microwave with a power of 1 W at the frequency of 5, 9.76, 10, and 15 GHz was excited from the port Ⅰ and injected into the MAM along the z-axis (the direction of electric field polarization 5 and magnetic field polarization was along the x-axis and along the y-axis, respectively). Perfectly matched layers (PML) at the top of the computational domain are applied to absorb the reflected and transmitted microwaves, respectively.

2.4. **Conductivity Test**

The conductivity of composite materials was measured using a digital multimeter (Tektronix DMM6500). Electrical conductivity was calculated according to the following equations, where L is the length of the test sample, S is the cross-sectional area of sample and R is the resistance of sample).

$$\sigma= \frac{L}{SR}$$

(S13)

2.5. **Joule Heating Test**

The IV curve of the composite was measured by electrochemical workstation (CHI660D). To investigate the thermal energy conversion performance, the foam sample was connected to a power supply (UTP-1310, UNI-T Co., China) at constant voltage for Joule heating tests.

2.6. **Photothermal Conversion Test**

A 500 W Xenon arc lamp was employed to simulate solar radiation. The incident light intensity was calibrated by adjusting the distance from the lamp to the sample. The aerogel was mounted on a thermally insulating glass substrate to prevent heat dissipation. Real-time surface temperature monitoring was conducted using an IR thermal imaging camera (FLIR, E6) positioned at a 30° incidence angle.

**Figure S1.** Photographs of the 4 mg mL^−1^ FGO and GO dispersions (left) and their hydrogels reduced by ascorbic acid at 80 °C for 12 hours (right).

**Figure S2.** Photographs of GF and SPGA.

**Figure S3.** The density of GF and SPGA.

**Figure S4.** POM images of FGO dispersion with (a, d) 40 μL, (b) 80 μL, and (c, e) 120 μL APG content. (f) SEM images of microstructures of the SPGA-2.

**Figure S5.** The SEM image of GF.

**Figure S6.** SPGA rests on the flowers.

**Figure S7.** Stress retention of SPGA-2 during 50 cycles at 70% compressive strain.

**Figure S8.** R and A coefficients of GF.

**Figure S9.** MA performance of SPGA. (a) SPGA-1, (b) SPGA-2, and (c) SPGA-3.

**Table S1.** Comparison of the minimum RL with those of other graphene-based foam absorbers reported previously.

| Sample | EAB (GHz) | RL_min_ (dB) | Ref |
| --- | --- | --- | --- |
| ZnONWs/RGO/PDMS | 4.20 | –27.8 | ^1^ |
| 3D rGO-CNT-Fe_3_O_4_ | 5.7 | –50.5 | ^2^ |
| Co_3_O_4_/Reduced graphene oxide/Melamine derived carbon foam | 3.4 | –31.88 | ^3^ |
| NRGO/ZnFe_2_O_4_ | 5.4 | –40.2 | ^4^ |
| Fe_3_O_4_ hybrid nanoclusters/frGO | 6.96 | –35.63 | ^5^ |
| aligned Ti_3_C_2_T_x_ MXene/RGO | 4.39 | –26.79 | ^6^ |
| CoFe_2_O_4_/N-doped reduced graphene oxide | 6.48 | –60.4 | ^7^ |
| SC-rGO | 4.4 | –53.68 | ^8^ |
| This work | 7.7 | –59.44 | **This work** |

**Figure S10.** (a) CST simulation results of (b) the 3D RCS plot for the PEC.

**Figure S11.** CST simulation results of the 3D RCS plot for the PEC layer covering with (a) GF, (b) SPGA-1, (c) SPGA-2, and (d) SPGA-3. (e,f) Corresponding RCS curves and values (g) for the PEC layer covering with GF and SPGA.

**Figure S12.** The (a) *μ′* and (b) *μ″* of GF and SPGA.

**Figure S13.** The Cole-Cole curve of (a) SPGA-1, (b) SPGA-2, and (c) SPGA-3.

**Figure S14.** The impedance matching characteristics of GF.

**Figure S15.** (a) SEM images, (b) conductivity at various compression ratios, *Z* under (c) 0, (d) 20%, (e) 40%, (f) 50%, (g) 60% and (h) 70% compressive strain, respectively of freeze-dried RGO aerogel.

**Figure S16.** (a-e) 2D RL mapping of SPGA-1 under 20%, 40%, 50%, 60%, and 70% compression strains.

**Figure S17.** EM parameter of SPGA-1 at different compression strains. (a) *ε′*, (b) *ε′′*, (c) *μ′*, (d) *μ′′*, and (e) *α*.

**Figure S18.** (a) conductivity of SPGA-1, 2D Z mapping of (b) 0%, (c) 20%, (d) 40%, (e) 50%, (f) 60%, and (g) 70% compression strains.

**Figure S19.** Dynamically adjustable MA performance of SPGA-1 at different compression strains with an initial thickness. RL curves at initial thickness of (a) 4 mm, (b) 5 mm, (c) 6 mm, (d) 7 mm, and (e) 8 mm.

**Figure S20.** (a-e) 2D RL mapping of SPGA-2 under 20%, 40%, 50%, 60%, and 70% compression strains.

**Figure S21.** (a-e) 2D RL mapping of SPGA-3 under 20%, 40%, 50%, 60%, and 70% compression strains.

**Figure S22.** Dynamically adjustable MA performance of SPGA-3 at different compression strains with an initial thickness. RL curves at initial thickness of (a) 3.63 mm, (b) 5 mm, (c) 6 mm, (d) 7 mm, and (e) 8 mm. EAB traces at initial thickness of (f) 3.63 mm, (g) 5 mm, (h) 6 mm, (i) 7 mm, and (j) 8.0 mm.

**Figure S23.** Dynamically adjustable MA performance of SPGA-3 at different compression strains with an initial thickness. (a) RL and (b) corresponding effective absorption bandwidth (EAB) values at 3.6−8 mm.

**Figure S24.** CST simulation results of PEC (a,b) and SPGA-2 under 0, 20, 40, 50, 60%, and 70% compression strains (c-h). (i) Total corresponding RCS curves.

**Figure S25.** EM parameter of SPGA-2 at different compression strains. (a) *μ′* and (b) *μ′′*.

**Figure S26.** The Cole-Cole curves of of SPGA-2 under (a) 0%, (b) 20%, (c) 40%, (d) 60%, and (e) 70% compression strains.

**Figure S27.** 2D Z mapping of SPGA-2 under (a) 0%, (b) 20%, (c) 40%, (d) 50%, (e) 60%, and (f) 70% compression strains.

**Figure 28.** The *t_m_* at quarter-wavelength model of SPGA-2 at different strains. (a) 0%, (b) 20%, (c) 40%, (d) 60%, and (f) 70%.

**Figure S29.** Simulation of PLD distribution at 6.32, 7.68, 10.72, 12, 14.08, and 16.08 GHz, under 20% and 40% compressive strain, respectively.

**Table S2.** The power loss density maximizes at 6.32, 7.68, 10.72, 12, 14.08, and 16.08 GHz, under 20% and 40% compressive strain, respectively.

| compressive strain (%) | PLD at  6.32 GHz  (MW m^－3^) | PLD at  7.68 GHz (MW m^－3^) | PLD at  10.72 GHz (MW m^－3^) | PLD at  12.00 GHz (MW m^－3^) | PLD at 14.08 GHz (MW m^－3^) | PLD at 16.08 GHz (MW m^－3^) |
| --- | --- | --- | --- | --- | --- | --- |
| 0 | 11.4575 | 9.3480 | 6.6008 | 6.54856 | 5.9446 | 5.6074 |
| 20 | 11.5738 | 15.8763 | 11.7375 | 10.7373 | 9.3588 | 8.7375 |
| 40 | 12.0343 | 19.2794 | 22.4582 | 20.2053 | 17.6266 | 14.6282 |
| 50 | 6.5485 | 10.7373 | 20.2053 | 25.0116 | 30.9433 | 10.8941 |
| 60 | 11.7972 | 17.7045 | 30.1917 | 30.9433 | 31.9641 | 28.3973 |
| 70 | 8.8762 | 9.1029 | 9.1718 | 10.8941 | 12.9063 | 17.1053 |

**Figure S30.** Joule heating performance of SPGA-2. (a) *I-V* curves of SPGA, (b), (c), and (d) Time-dependent surface temperature of the SPGA-2 with various supplied voltage. (e) Time-temperature curve of SPGA-2 at 3.5 V constant voltage. (f) *I-V* curves of SPGA-2 at different compression strains. (g) and (h) Time-dependent surface temperature of the SPGA-2 at different compression strains at 2.0 V constant voltage. Photographic images of (i) de-icing and (j) electro-thermochromic process of SPGA-2 at a voltage of 2 V.

The weakly strain-dependent conductivity of SPGA suggests that the same spherical-pore topology which stabilizes EM impedance may also suppress thermal runaway in resistive heating. *I–V* curves of SPGA-2 exhibit linear, Ohmic behavior over the tested voltage range (**Figure S30a**), indicating that Joule heating is dominated by inelastic electron–phonon scattering. Under low operating voltages (1.5–3.5 V), the surface temperature (*T*_s_) of SPGA-2 rises rapidly and reaches a steady state within ≈50 s, and drops back to ambient almost immediately after the power is switched off (**Figure S30b**). *T*_s_ increases nearly linearly with applied voltage, reaching ≈190 ^o^C for SPGA-2 at 3.5 V (**Figure** **S30c**), and cyclic heating/cooling as well as long-term operation confirm highly stable and reproducible thermal output (**Figure S30d,e**). *T*_s_ scales linearly with applied voltage, reaching 190 °C for SPGA-2 and 210 °C for SPGA-3 at 3.5 V (**Figure S31**).

Under compressive strain, SPGA maintains this stable Joule heating behavior. *I-V* curves remain linear at all tested strains (**Figure S30f**), and *T*_s_ at a fixed bias shows only modest changes with increasing compression. At 2.0 V, *T*_s_ increases by only ≈24 °C at 50% strain and by ≈85 °C even at 70% strain (**Figure S30g,h**), demonstrating that large mechanical deformation does not trigger catastrophic overheating. This strain-insensitive heating behavior directly reflects the topology-controlled, slowly varying conductivity–strain relationship, and confirms that the spherical-pore architecture, which moderates impedance evolution in microwave absorption, also suppresses thermal runaway under mechanical perturbation. In practice, SPGA-2 can rapidly de-ice and enable electro-thermochromic camouflage at 2 V (**Figure S30i,j**), illustrating its potential in safe, deformable thermal-management devices.

**Figure S31.** Time-dependent surface temperature of the SPGA-3 with various supplied voltage.

**Figure S32.** Photothermal conversion performance of SPGA-2. (a) UV–Vis–NIR absorption spectra of SPGA. (b, c) Surface temperature variations of SPGA-2 with different light irradiation. (d) A linear fitting correlation between the saturated temperature and light intensity. (e) Thermal response of SPGA-2 switching at 100–300 mW cm^−2^. (f) The time–temperature curve at long-term light irradiation of 300 mW cm^−2^ for SPGA-2. (g) Photographic and infrared thermal images of SPGA-2 under outdoor sunlight at 28 °C heating up and melting 2 cm^−3^ of an ice cube.

In extreme environments, SPGA enables dual-energy defogging and de-icing through both electrical Joule heating and solar-thermal conversion, the latter offering an environmentally benign energy pathway. As shown in **Figure S32a**, SPGA exhibits strong absorption across the visible spectrum (400–800 nm).^[56]^ Under simulated solar illumination, SPGA-2 displays rapid photothermal response: at 100 mW cm⁻², the surface temperature *T*_s_ quickly rises to ≈82.9 °C and returns to ambient upon light removal (**Figure S32b**). Increasing the intensity to 300 mW cm⁻² elevates *T*_s_ to ≈172 °C. Remarkably, SPGA-2 achieves ≈86% of its steady-state *T*_s_ within just 10 s, corresponding to a nearly 120 °C rise under 300 mW cm⁻² (**Figure S32c**). It further achieves an exceptionally high heating rate of up to ≈12 °C s⁻¹, representing a ≈21-fold improvement over the previously reported 0.57 °C s⁻¹ for para-aramid pulp/graphene aerogels.^[57]^ This ultrafast response, coupled with the linear scaling of *T*_s_ with light intensity (**Figure S32d**), ensures precise and predictable thermal control. Stability, a critical factor for practical deployment, was systematically validated: optical heating–cooling cycles (100–300 mW cm⁻²) reveal negligible performance degradation over multiple runs (**Figure S32e**), while prolonged irradiation (600 s) confirms excellent thermal durability (**Figure S32f**). The real-world utility of SPGA-2 is further demonstrated in outdoor testing. Under natural sunlight at ≈28 ^o^C ambient conditions, *T*_s_ increases by ≈37 ^o^C, reaching ≈65 ^o^C within only 8 s (**Figure S32g**). This rapid solar heating enables efficient de-icing, fully melting a 2 cm³ ice cube within 4 min, where passive thawing is ineffective. Collectively, the rapid response, tunable heating, and long-term stability of SPGA establish it as a versatile and reliable thermal-management material. Together with its programmable EM absorption, these results underscore that the spherical-pore topology offers a unified structural strategy for multi-field adaptive protection in harsh and dynamic environments.

**References**

[1] Song, X. Yin, M. Han, X. Li, Z. Hou, L. Zhang, L. Cheng, *Carbon* **2017,** *116*, 50-58.

[2] J. Li, Y. Xie, W. Lu, T.-W. Chou *Carbon* **2018,** *129*, 76-84.

[3] Y. Li, S. Li, T. Zhang, L. Shi, S. Liu, Y. Zhao, *J. Alloys Compd.* **2019,** *792*, 424-431.

[4] R. Shu, J. Xu, Z. Wan, X. Cao, *J. Colloid Interface Sci.* **2022,** *608*, 2994-3003.

[5] Y. Kang, W. Li, T. Ma, X. Huang, Y. Mo, Z. Chu, Z. Zhang, G. Feng, *Compos. Sci. Technol.* **2019,** *174*, 184-193.

[6] Y. Ning, M. Yang, Z. Zhao, X. Sun, S. Yang, S. Wang, L. Liang, Y. Cheng, W. Yin, Y. Yuan, Y. Li, *Compos. Sci. Technol.* **2022,** *227*, 109609.

[7] X. Wang, Y. Lu, T. Zhu, S. Chang, W. Wang, *Chem. Eng. J.* **2020,** *388*, 124317.

[8] X. Deng, S. Gao, Y. Liu, Y. Bao, Y. Zhu, Y. Fu, *Appl. Surf. Sci.* **2022,** *599*, 154063.
